# Abstracts and Gleanings

**Published:** 1883-05-20

**Authors:** 


### ABSTRACTS AND GLEANINGS.

**Colic in Children.**—The Medical Times and Gazette says: In a clinical lecture delivered by Hofrath Prof. Widerhofer, and reported in the Allg. Med. Zeitung, No. 22, we find the following observations:

By the term colic we understand an intestinal neurosis originating in irritation of a chemical or mechanical kind, of the sensory nerves of the mucous membrane of the intestinal canal. There may also occur purely nervous colic, wherein neither irritating ingesta nor a pathological state of the affair is present, excitement of the central organs being propagated to the nerves of the canal. In infants who are at the breast it is indigestible milk, and especially when this is too rich in fatty matters, that causes the colic; and when children during the first six months are fed with amalaceous food, before a sufficiency of saliva is secreted, colic is also produced. This occurs, too, when indigestible matters are swallowed, such as sand, small pebbles, etc.; and we have good opportunities of observing the operation of this cause in idiots, who often swallow such objects in great numbers. And here we have to meet the question, whether during the period of lactation the mental emotions of the nurse may not induce colic in the infant. It is beyond doubt that frequent mental emotions may induce colic with convulsions, which may be explained by the changes that are induced in the secretion of the milk. In the group of colics induced by irritation caused by the contents of the canal, must be included that caused by constipation, by worms and by the presence of foreign bodies. Of the morbid conditions of the mucous membrane which give rise to colic, enteritis folliculosa may be especially mentioned, and then scrofulous and catarrhal ulcers, the worst forms being observed in intussusception. Pure nervous colic appears in diseases of the spinal cord, and it may appear in hysterical form, which is not so very rare, and also as intermittent colic, with a regular rythm as intermittent fever. We may also include metallic colic, which certainly occurs far more frequent in children than it is diagnosed, as might be expected from the frequency with which toys are made of or contain lead. As regards dignosis, the purely windy colic producd by the collection of gases which distend the canal and irritate the sensory nerves, comes on with distention of uthe abdomen, ending with the expulsion of flatus. These attacks are paroxysmal, and are frequently accompanied by clonic convulsions, which may last for some minutes, and even for an hour or more. After the cessation of the paroxysm the child is either itself again or may remain dull and feeble. In the intervals of the attacks there are no essential cerebral symptoms perceptible. The prognosis depends upon the nature of the cause, but it has been questioned whether a colic of itself may prove fatal. Through the long duration of the accompanying convulsions, through the shock and the exhaustion of the nervous system, death may follow, and at the post-mortem no anatomical cause of the fatal termination can be shown. Hysterical attacks of the colic especially concern very excitable children, nervous girls, and are characterized by violent pains, a drawn-in abdomen, slight convulsions, and obstinate constipation. In the treatment of colic, we must first endeavor to remove the cause. In suckling infants, colic is especially apt to occur when the nurse's milk exhibits a large proportion of fat, and in such a case the nurse should be changed. In flatulent colic, oleum chamomilæ or fœniculi may be given, with a drop of tincture of opium, as oleo-saccharate. In metallic and in hysterical colic, belladonna is the best means; and intermittent colic should be treated by quinine.—*Medical and Surgical Reporter.*

**Treatment of Spermatorrhœa.**—Obstinate cases of spermatorrhœa and frequent nocturnal emissions constantly come under the care of the practitioner. Too frequently the medical man consulted simply tells the patient that if he breaks off the pernicious habit of masturbation, which has probably originated his malady, he will quickly recover. But in fact, in most cases, the habit has already been abandoned before he came to seek advice; and these cases do not get well for months or even years afterward, unless proper measures be taken. Knowing that he has left off this bad habit, and that he nevertheless does not improve, his complaint being made light of by the regular practitioner, and being greatly depressed in mind, he seeks the advice of the quack, who is always ready to benefit by these cases. I will give an outline of the treatment I have followed, and which I have found most successful in several such cases. The treatment should be, (1) Moral, (2) Hygienic, (3) Medicinal.

1. *Moral.*—(*a*) The pernicious habit of masturbation, which has probably been the origin of the complaint, must at once be discontinued, or no good can result from any treatment. (*b*) The thoughts should be directed from himself by his having regular work and exercise. (*c*) The anxiety of mind which ensues should be allayed as much as possible and a happy state of mind instituted.

2. *Hygienic*—(*a*) The patient should have regular but not excessive mental employment, and bodily exercise in the form of walking, riding, or out-door sports and games. (*b*) Cold sponging of the genitals night and morning for some minutes, or as long as can comfortably be borne, is a most important agent in giving tone to the relaxed organs. (*c*) The patient should have a hard mattress, and as little and light clothing as possible at night. Care should be taken not to lie on the back, which may be prevented by wearing a knotted towel over the spine, or by some other device. (*d*) No quantity of liquid should be taken before retiring to rest, and the bladder should be emptied the last thing.

3. *Medicinal*—A mixture containing tincture of perchloride of iron and tincture of nux vomica should be given twice or three times a day; also a pill containing a fourth or a third of a grain of extract of belladonna with three grains of camphor should be given at first every night, and then every other night, immediately before going to bed. If these lines of treatment be adhered to, the patient, whether suffering from real spermatorrhæ or simply from frequently returning nocturnal emissions, will steadily improve, and the emissions will occur less and less frequently, till, in the course of a few weeks, or possibly months—for a malady of long standing (as this usually is) is never cured immediately—they will cease altogether, or only occur at such intervals as may be deemed normal, and in which there is no harm whatever.—*Boston Medical Journal.*

**Poisoning by Carbolic Acid.**—Ruge (Berlin. Klin. Woch., Oct. 30, 1882, and Lond. Med. Rec., March 15, 1883), relates the case of a woman, æt. 59, who took by mistake for a dose of medicine a tablespoonful of concentrated solution of carbolic acid (95 p. c.). The whole quantity was swallowed. The instantaneous effect was a fearfully intense sensation of burning in the mouth and throat. The face became pale, the hands and feet cold and pulse scarcely perceptible. Vomiting did not ensue, so that the whole dose was retained. Fortunately antidotes were immediately procured. First, the patient drank freely of milk, and then of a mixture of milk, the white of an egg and carbonate of magnesia. After taking these, vomiting occurred. The white of the egg was returned in lumps like hard-boiled egg and the vomited matters had no smell of carbolic acid. The above treatment was continued for several days. It is remarkable that no special signs of the poisoning supervened. The urine voided was dark but free from albumen and soon became normal. No fever ensued. The interior of the mouth was corroded and very painful, the pain extending far down the œsophagus. The epigastrium was not tender to pressure. The mucous membrane of the mouth and tongue came away in shreds and large quantities of mucus originating doubtless in the œsophagus, were rejected by vomiting. The severe pain in swallowing continued six days, the patient being able to take only pultaceous food. Dysphagia slowly diminished, but had not enirely disappeared September 27, (the acid was swallowed August 23), solid food requiring a greater effort of digestion. In all other respects the patient was perfectly restored to health.—*Maryland Med. Journal.*

**An Insect that Secretes Prussic Acid.**—It has often been noticed by gardeners and others that some kinds of centipedes when caught, or otherwise irritated, emit an odor of prussic acid. G. Geldensteeden has shown that the insects do absolutely contain prussic acid, and that the acid is contained in glands in the skin, which lie symmetrically on both sides of thecreature. The glands are situated in the adepose tissues, are of an eliptical shape, and about 5 mm. long.

M. DUMAS recommends water saturated with alum for use in extinguishing fires, and the French Minister of the interior has recommended that firemen should be supplied with facilities for using alum.

**Dyspepsia.**—Dr. Blackwood, in Medical Times, recommends the following "shot-gun" prescription in dyspepsia from hepatic derangement:

℞ Cinchonidiæ sulphatis........................ } 
Euonymin .............................. ....
Irisin.........................................
Leptandrin.................................
Juglandin, aa.............................. ʒ ss.,
Podophyllin...............................
Ext. belladonnæ..........................
Ext. nucis vomicæ...........................
Ext. hyoscyami,................................ gr. x.
M. In pill, No. 60 div. Sig. One or two at bed-time.

Many stubborn cases of dyspepsia, that ran the gauntlet unavailingly of all sorts of peptonoids, has given way to this, and it is an admirable cholagogue on general principles. In scrofulous subjects, with deficient nutrition, I have had much benefit from minute doses of mercuric bichloride (the one-hundredth of a grain) in tinct. calumbæ comp, the dose being a drachm of the latter three times a day. Within a few weeks a most interesting case, treated by several physicians for organic cardiac lesion, has recovered under the remedies just alluded to. The palpitation, the supposed dilatation with compensating hypertrophy according to canonical dicta, has subsided; the patient can lie down, and sleep too, when incumbent; he has no night tremor or dread; he can eat, drink, and be merry now; whereas, before he was morose, taciturn, and a family nuisance; in short, he has dropped a minor dyspepsia, and with it a prognosed incurable heart-trouble. Dyspepsia, like charity, covers a multitude of troubles and sins, and a good deal of the "malaria" so fashionable with the fraternity, and with the laity also, is one or another form of indigestion.

**Typhoid Fever.**—A writer in Brief, relies upon turpentine in the treatment of typhoid fever as follows:

℞ Sugar.................................. 2 drachms,
Gum arabic.............................. 2 drachms,
Ol. turpentine........................... 2 drachms.

Mix and thoroughly triturate in a mortar, and during the process slowly add four ounces of cinnamon water. Sig. One teaspoonful every four hours.

We allow the patient a bountiful supply of good, cold buttermilk, religiously avoiding those most innocent articles, beef-tea and chicken-water, while true as steel to all the laws of hygiene. If cordials are indicated, egg-nog, with the egg always left out. The dose is for an adult, to be graduated to the ages of children.

As soon as we ascertain a fever submitted to our care to be typhoid, we immediately resort to this medicine. If the patient has diarrhœa, we look for the number of discharges to diminish every day till it ceases—opium for the loose bowels always produces head symptoms of a most unpleasant character.

**Electric Light in Surgery.**—Mention was long since made in our columns of some curious experiments that have been made in Europe in lighting up internal cavities of the body by means of electricity, with a view to enable the physician better to "see into" the case. This method of exploration seems likely to become no novelty in surgery. Apparatus is now being made in Vienna for illumnating the throat, nasal passages, bladder, and other portions of the inner man. "Let daylight shine through" a person is an old idea, but the rendering the body transparent and making plain hidden recesses is a different thing.

Dr. Thomas Oliver, in an English medical journal, refers as follows to his own experience with this application of electricity:—

Having at the present time a patient in the infirmary who is suffering from hydatid disease of the liver, on whom the operation of abdominal section with incision of the liver had been performed, giving exit to about seven pints and a half of pus—I took advantage of the opportunity, and succeeded in lighting up the interior of the cyst by means of the electric light. For this purpose Mr. Payne devised and constructed a brass tube, electro-plated, nine and a half inches length, and eleven-sixteenths of an inch diameter externally. One end of this tube was funnel-shaped, and the other was closed by a piece of glass; down this tube was inserted a narrow cylinder, which carried a Swan's lamp and the electric wires. This tube, with its glazed extremity, was smeared with carbolized oil, although, in future, I shall use carbolized glycerine for the window of the tube, and, with gentle pressure, I succeeded in passing through the abdominal incision into the liver. The lamp was at once lit, and I had the pleasure of observing a greyish-red condition of the wall of the cyst, studded across which were numerous yellow white spots, evidently pus; a slight oozing or sweating, was also noticed on the wall of the cavity. The illumination of the interior of the liver by means of the electric light was in every way satisfactory and successful; and although it is of little aid in the treatment of the case in question, it has shown us that the lighting up of internal cavities is now not only a possibility, but a matter of comparative ease. With the extremely small size of Small's lamp required (it is not much larger than an ordinary bean) which gives a light equivalent to that from candlers, and with the improved instrument which Mr. Payne is devising, I see how the electric light might become useful in operations for vesico-vaginal, or recto-vaginal fistula, and in certain diseases of the bladder.

**Treatment of Placenta Pvævia.**—Dr. Hofmeir, assistant physician at the University Gynæcological Department of the University of Klinkik, met with forty-six cases of placenta prævia in a little over one year. His experience has led him to reject the expectant plan of treatment, and we will not withold our congratulations on his arriving at such a happy decision. Thirty-seven out of forty-six cases were treated actively from the moment operative treatment was practicable. He did not wait till the cervix *was dilated.* The manual method of turning was employed thirty times; in three of the cases a foot was already down; three times internal version was performed; and in one forceps were used. It was observed in every case that the hemorrhage ceased whenever traction was employed on the fœtus. He considers that the principle of earliest possible intervention should not be departed from, even when the cervix is contracted and the external os tolerably small. In these cases the finger should be thrust through regardless of placenta, and a foot drawn down. When the placenta is centrally situated, or whenever the hemorrage is copious, the danger to the mother is so great that danger to the child should not be brought in comparison with it. When once a foot is down, however, there is no longer any need for haste. On the contrary, too much speed may now bring about the very danger it has been the attendant's object to escape from. Thus, rapid delivery now—too rapid delivery—might and would, cause laceration of the cervix; and this, in the bruised and wounded condition of the part, would undoubtedly be the cause of serious, harassing, and perhaps fatal hemorrhage. For these reasons, then, extraction, after a foot is once down, should be slow. Hofmeir is in the habit of injecting subcutaneously 0.4 gramm. of ergotine, and after a delivery syringing out the uterus with a 5 per cent. carbolic solution. The results obtained were just what might be expected when early and rational treatment is adopted. One death only occurred in the thirty-seven cases treated in the above manner, and in the fatal case death took place on the seventeenth day from pneumonia phlebitis; she had, moreover, been treated by tampons for twenty-four hours before labor. These results compare well with those usually met with in placenta prævia —viz, a mortality of from 30 to 40 per cent. Of the thirty-seven children, seventeen were premature children, and three died in consequence of the perforation of the placenta. Perhaps Dr. Hofmeir is hardly fair to himself. The danger to the child from deprivation of oxygen is in placenta prævia so great that it is more than probable that a larger proportion of infants would have died had any other method of treatment been followed out.—*Med. Press.*

**A Needle Five Years in the Body.**—Case I. Mrs. W. in 1830 swallowed a large needle with a broken point. Considerable irritation occurred, the needle apparently lodging, and attempts to remove or force it down caused some vomiting of blood, and for two weeks afterward she brought up blood from the throat. About twenty years later she was seized suddenly while stooping with intense lancinating pain in the left hip joint, which made movement agonizing, and confined her to bed several weeks. Recurrence of this pain took place twice, at intervals of one and two years, in each instance the attack coming on suddenly while using the limb—always in the left hip. In 1874 she was instantly and violently attacked in the left shoulder and arm with an exquisite pain, worse even than the worst rheumatism. This was attended by more or less swelling, and was considered rheumatic, but resisted all treatment. It lasted several months, and disappeared of itself, and for nearly two years she experienced no trouble. But in 1876 she was again similarly attacked, this time with a severe stinging pain with redness and swelling in the posterior aspect of the left arm, three inches above the elbow joint. All remedies failed to effect relief, when in applying a liniment, something wounded her hand, and on looking for the offending object a blunt needle-point was discovered, but so firmly held in its location that an incision was required, and considerable force was necessary to extract it with forceps. The needle was blackened, and had lost its smoothness. Careful examination showed the needle to be one of an ancient pattern which had long since ceased to be manufactured. Since removing the needle the lady enjoys perfect immunity from pain and is now a woman of eighty, with the physical and mental vigor of one of forty years of age.—*Southern Medical News*.

**A New Treatment of Dysentery.**—Dr. F. Rawle recommends the following treatment in the British Medical Journal, January 27, 1883:

First, having placed the patient between warm blankets, I proceed to inject a pint and a half of warm water, at a temperature of 90° Fahr. This is seldom retained longer than a few minutes, but is pronounced very grateful to the patient. When the water has soothed the mucous membrane of the colon and rectum, and brought away any *effete* matter, I then proceed to administer a small injection of two ounces, by measure, with a gum-elastic bottle. The form I administer is the following:

| | | |
|---|---|---|
| ℞ | Quiniæ disulphat. | gr. x, |
| | Tinct. camphoræ comp. | ʒ iv, |
| | Decoctum amyli ad | ℥ ij. |

M., and when about milk-warm inject.

It is generally retained, but if ejected, it may be repeated after an hour or two. This I found of great service, and very grateful to the patient. I do not stop to inquire how it acts, but the effect is like magic. If griping pains be felt over the region of the epigastrium, I administer half-drachm doses of chlorodyne, in some aromatic water, mint, caraway, or aniseed. The diet, of course, should be of the most soothing kind; jellies, isinglass, linseed, toast and barley-water, *ad libitum*. Ipecacuanha I have found of little service, and have discarded it from my treatment. If any of my medical brethren will try these measures, he will not often be disappointed. I have used with advantage warm turpentine stupes on warm flannels, over the hypogastrium.—*Med. and Surg. Reporter*.

**A Prognostic Sign of Pneumonia.**—Dr. J. B. Sullivan, of Stanton, Michigan, contributes the following:

I have had considerable experience in the treatment of pneumonia, and have realized, as every practitioner must, that it is a formidable disease. I think I have detected a symptom which, when discovered, indicates an unfavorable prognosis, and the absence of which justifies a promise of recovery. I have relied on it for twenty years. In a case of typical pneumonia we have five stages, viz: engorgement, red hepatization, gray hepatization, suppuration and resolution. Dr. Stokes describes a stage of arterial injection, before engorgement, but I am content with regarding this as the first stage. Engorgement is congestion of the pulmonary vessels. During red hepatization the lung has a dull reddish-brown tint, and in this stage the sputa will reveal a breaking down of the lung substance, if such destruction is taking place. The pleura almost invariably participates in the inflammatory changes when the superficial portion of the parenchyma is affected. When red hepatization has existed for some days (as it usually does) the color becomes paler and whiter. Gray hepatization succeeds the red and its occurrence may be detected by the color of the sputa. It is at the onset of this stage that we have our sign. If the stage of red hepatization, as indicated in the characteristic reddish sputa, do not continue for at least thirty hours, the patient will die. This has been my experience. Practical physicians make a note of it, and report your observations in the Age.—*Med. Age.*

**Quinine in Whooping-Cough.**—Dr. Parker, in Medical and Surgical Reporter, says of the quinine treatment:

I give a teaspoonful of a solution of sulphate of quinine four, six, eight, or even ten grains to the ounce, oft repeated. This remedy does not disappoint in many cases in controlling the disease, and if properly used, and with perseverance, in actually curing it, or at least, shortening its course very decidedly. It seems to act as a destroyer of the fungi. It also nauseates and loosens the mucus in which they exist, and has also the valuable properties of a tonic. Unlike many of the other remedies which are so unsuccessfully exhibited in the disease, it has absolutely no injurious effects. The little patients begin to improve very shortly after the first two or three doses. I am fully convinced that a trial should always be made of the solution of sulphate of quinine in the strength and in the doses indicated, according to the age of the patient and the severity of the case; and after a few faithful experiments in this direction, no one will be able to say with truth that "the course of the disease could not be controlled by treatment."

**Hypertrophy of Tonsils—Interstitial Injections.**—Professor Moresco, of Cadiz, read a paper before the Congress of Seville (Revista de Med. y Cirurgia pratica) in which he recommended the treatment of hypertrophy of the tonsils by interstitial injections of acetic acid: he reports two cases perfectly cured by this method. He gave the following as the advantages of his method:

1. Its facility of performance.
2. The impossibility of causing any serious results.
3. The gland preserves its functions.
4. It requires no interference with the patient's occupation,
5. It is absolutely painless.—*Rev. Mems. de Laryngol., d' Otol. et de Rhinol.*—*Med. News.*

**Bi Carb Soda for Burns.**—The application, a writer in *Popular Science Monthly* says: "All that is necessary is to cut a piece of lint or old soft rag, or even thick blotting paper, of a size to cover the burned or scalded parts, and to keep it constantly well wetted with the sodiac lotion as to prevent its drying. By this means it usually happens that all pain ceases in from a quarter to half an hour, or even in much less time. When the main part of a limb, such as the hand and forearm or the foot and leg, has been burned, it is best, when practicable, to plunge the part at once into a jug or pail, or other convenient vessel filled with soda lotion, and keep it there until the pain subsides; or the limb may be swathed or encirled with a surgeon's cotton bandage previously soaked in the saturated solution, and kept constantly wetted with it, the relief being usually immediate, provided the solution be saturated and cold. What is now usually sold as bicarbonate of soda is what I have commonly used and recommeded, although this is well known to vary much in quality, according to where it is manufactured—but it will be found to answer the purpose, although, probably, Howard's is most to be depended on, the carbonate being too caustic. It is believed that a large proportion of medical practitioners are still unaware of the remarkable qualities of this easily applied remedy, which recommends itself for obvious reasons."

**Greasing with Fat Bacon for Scarlatina.**—Silas Hubbard' M. D., in Peoria Medical Monthly, writes:

In the March number of this journal Dr. J. M. Hole gives his successful experience of treating scarlet fever with inunction of hog lard. I would say that for more than thirty-five years I have treated many cases of scarlatina in part by frequently greasing the patient with fat bacon with satisfactory results.

Dr. Merrill contended that the good effect of lard inunction was that the fever spent itself upon the lard instead of the fat of the patient. I supposed that the good effects of the grease was either by killing the scarlatina bacteria or furnishing them food until they died a natural death.

So far as I know my brain originated the supposition that scarlatina is an attack of millions of parasites on the human subject, and to fight them I greased the patient, and recently have in a number of cases given frequent small doses of sulphur morning and evening to the well in the family afflicted with the disease, as a prophylactic. The patients recovered speedily, and the well did not take the disease, or had it lightly.

**Mi..eral Acids in Summer Diarrhœa.**—The mineral acids are very efficient in sporadic cholera and summer diarrhœa. The indications for their use are the profuse and watery character of the discharges, which are alkaline or neutral in reaction, due to outward osmosis from the serum of the blood, and the best of the acids is sulphuric acid given with opium. Hope's camphor mixture is also frequently used, especially in the pulmonary diarrhœa, with benefit.—*Bartholow.*—*Col. and Clin. Record.*

**Dr. Cattaneo's Treatment of Hydrocele.**—From the Jour. de Med. de Paris, we note the following treatment recommended by Dr. C.:

1. Puncture of the hydrocele with a capillary trocar of an aspirator, and evacuation of the fluid.

2. Injection of a solution of hydrate of chloral in quantity proportionate to the volume of the hydrocele and age of the patient; one to two grams of chloral for children, four grams for adults, and occasionally more in old men. The solution is made by dissolving equal parts of chloral in cold distilled water.

3. Cold applications to overcome the pain produced by the injection.

4. The injection is repeated if the absorption occurs too slowly.

The patients are kept in bed, and wear a suspensory bandage for some time after the termination of treatment. Dr. Lampagnani says that the effusion has not returned in any one of the seventeen cases operated upon by this method.—*Med. and Surg. Rep.*

**Perchloride of Iron in Skin Diseases.**—In the Rev. Clin. di Bologna. Dr. Carsarini thus sums up his experience :

1. Perchloride of iron is a most efficacious remedy in purpura hemorrhagica.

2. In the chloro-anæmia accompanying certain skin diseases—as rupia, eczema, impetigo, etc.

3. Its external use is very favorable in scrofulous and syphilitic ulcers.

4. Squamous affections are markedly modified by applications of a liniment of perchloride of iron.

5. It may be used as a lotion, dissolved in two or three parts of water, or as an ointment—one, two, or three grains of perchloride of iron to thirty grains of vaseline [cosmoline] or lard. The author has used it in psoriasis, in the form of a pomade—ten grains of iron, thirty grains of lard or glycerine.—*Med. and Surg. Rep.*

**Chromic** acid is the latest application commended as marvelously efficacious in syphilitic sores. We believe little in the specific propert es of local applications save when they act by absorption or as parasticides. What is well locally for syphilitic sores is good for other sores. Sometimes anodynes, sometimes stimulants, sometimes astringents, sometimes protectives, rarely escharotics, and never soap and water, are useful in healing sores. In a diphtheretic sore throat we have seen remarkable results from chromic acid applied to the diptheroid membrane, and should an opportunity offer we should try this acid on dipththeritic membrane in a solution of ten to sixty grains. For condylomata and other warty growths, chromic acid is excellent above all other remedies that we have tried. Salicylic acid in corns and hard warts is highly recommended.

**Quinine Nausea.**—Kaulich says that a few drops of tincture belladonnæ given before the ingestion of quininæ sulph. will surely preve..t vomiting.—*Lyon Med.—Can. Jour. Med. Sc*

**Belladonia in the Treatment of Hernia.**—Dr W. S. Batten, in British Medical Journal. says that in a case of Hernia in an old gentleman he was given half-dram doses of tincture of belladonna every half hour. In three hours the pupils were largely dilated; there was marked dryness of the throat, and the hernia was returned without difficulty. On a subsequent occasion, where the same condition existed in a slight degree in the same patient, three doses of the tincture of balladonna at half-hour intervals made it possible to reduce the hernia with facility.

The second case was that of a pale, pasty youth, aged nineteen, who had been ruptured from childhood. When lifting a heavy load the hernia came down by the side of the truss and could not be replaced. Taxis was attempted after the patient had been fully relaxed by being placed in a hot bath, and when under the influence of chloroform. Twenty minim doses of the tincture of belladonna, repeated every hour for four hours, made possible the reduction of the hernia.

**Special Indication for the Administration of Salicydate of Soda in Typhoid Fever.**—The London Med. Record, February 15, 1883, says that Bareggi (*Gazz. delgi Ospitalli*) Dec. 1883), having noticed in all cases acute articular rheumatism treated by him with salicylate of soda, that obstinate constipation occurred after two or three days' treatment, determined to utilize this action of the salicylate, in typhoid fever with profuse diarrhœa. He found it to answer admirably; the diarrhoœ ceased after two or three days, and the disease ran a favorable course. The salicylate may be given in larger doses than in rheumatism, without any bad effects on the digestive or nervous system.—*Quarterly Compendium.*

**M. Pasteur** has written from Vaucluse, where he now is, to the Medical Academy at Paris, to say that he has found the cause of the disease in pigs, which in the valley of the Rhone alone killed 20,000 animals lately. The disease is caused by a very minute microbe resembling that which causes cholera in fowls, but differs in physiological properties, being quite harmless to these latter animals, although it kills rabbits and pigs, particularly white ones. M. Pasteur succeeded in innoculating pigs with microbes obtained by artificial means, and thus preventing their ever having this disease.—*Medical Press.*

**Powdered Capsicum** as a remedy in sub-acute and chronic rheumatism has been recommended by Mr. A. Drummond McDonald in the "British Medical Journal." Two drachms to the ounce of lard, to which one of the essential oils may be added to make it more elegant, is the proportion mentioned. It is thoroughly rubbed over the affected part by a gloved hand for ten minutes at a time, night and morning, or at bed time only, according to the effect produced. Dry heat applied afterwards intensifies its effect, which lasts for sometime.—*Weekly Med. Review*, March 3, 1883.

